# Effectiveness, Feasibility, and Acceptability of Dynamic Elastomeric Fabric Orthoses (DEFO) for Managing Pain, Functional Capacity, and Quality of Life during Prenatal and Postnatal Care: A Systematic Review

**DOI:** 10.3390/ijerph16132408

**Published:** 2019-07-06

**Authors:** Jaclyn M. Szkwara, Nikki Milne, Wayne Hing, Rodney Pope

**Affiliations:** 1 Department of Physiotherapy/Faculty of Health Sciences and Medicine, Bond University, Gold Coast, QLD 4229, Australia; 2 School of Community Health, Charles Sturt University, Thurgoona, NSW 2640, Australia

**Keywords:** dynamic elastomeric fabric orthoses (DEFO), pregnancy, pre-natal, post-natal, postpartum, low back pain, pelvic girdle pain, compression garment, intervention

## Abstract

Conservative interventions for addressing prenatal and postnatal ailments have been described in the research literature. Research results indicated that maternity support belts assist with reducing pain and other symptoms in these phases; however, compliance in wearing maternity support belts is poor. To combat poor compliance, commercial manufacturers designed dynamic elastomeric fabric orthoses (DEFO)/compression garments that target prenatal and postnatal ailments. This systematic review aimed to identify, critically appraise, and synthesize key findings on the effectiveness, the feasibility, and the acceptability of using DEFO to manage ailments during pre-natal and postnatal phases of care. Electronic databases were systematically searched to identify relevant studies, resulting in 17 studies that met the eligibility criteria. There were variations in DEFO descriptors, including hosiery, support belts, abdominal binders and more, making it difficult to compare findings from the research articles regarding value of DEFO during prenatal and/or postnatal phases. A meta-synthesis of empirical research findings suggests wearing DEFOs during pregnancy has a significant desirable effect for managing pain and improving functional capacity. Further research is required to investigate the use of DEFOs for managing pain in the postnatal period and improving quality life during prenatal and postnatal care.

## 1. Introduction

Pregnancy-related ailments such as pelvic girdle pain [1], lower back pain [2], and vulval varicosities [3] are common concerns of women during pregnancy trimesters. As there is not a consensus that pregnancy-related low back and pelvic pain (LBPP) are separate conditions, they are regularly examined together [4]. Close (2016) [5] further supports this idea, suggesting that LBPP is multifaceted, with women reporting their pain levels as significant and using strong verbal descriptors such as “horrific” to describe their pain levels. Howell et al. (2012) [6] reported that the prevalence of pelvic pain in the prenatal phase can range between 48 and 71 percent. Research by Persson et al. (2013) [7] discovered key themes when women described their experiences of pelvic girdle pain, including that increased pain levels directly translated to an increased dependence on or need for support and assistance with activities of daily living including driving and cooking, a decreased satisfaction or pleasure during their pregnancy, an increased sense of “being a burden”, and an increased sense of “living with enduring pain” [5].

Prenatal complications can carry through to the postnatal phase, further complicating common postpartum conditions such as genital tract trauma, perineal pain, sexual problems, incisional pain, and urinary incontinence [8,9,10,11]. More recently, Howell et al. (2012) [6] indicated that self-reported high intensity of pain during the prenatal phase strongly correlated to the earlier onset of pelvic pain in the postnatal phase with rates of reoccurrence ranging from 41–77%.

As with pregnancy-related ailments, postpartum complications from vaginal deliveries and caesarean sections are common and can cause women notable pain and discomfort [12]. Common general complaints reported during postnatal hospital stays include extreme tiredness and exhaustion, painful perineum, breast problems, constipation, and back pain [12]. More specifically, vaginal deliveries (either spontaneous delivery or instrumental delivery) are often associated with additional complications that can cause pain to women, detrimentally affecting their activities of daily living [9,10]. Caesarean section is considered a major abdominal surgery and is a common surgical procedure that is on the increase due to the number of maternal requests without obstetric indication [8]. Some of the major complications following caesarean section are persistent low back pain not associated with epidural or spinal anesthesia [11], a higher risk for severe pelvic-girdle syndrome at six months postpartum [8], and incisional pain [11]. Since caesarean sections are considered major abdominal surgery, it is important to note that, in addition to population specific complications, women who have had a caesarean section also need to address problems such as wound management, healing timeframes, bed mobility, risk of deep vein thrombosis [13,14], strength and muscle activation, reactivation of the “core” abdominal muscles after surgical incision [11,12], and the effects of immobilization on muscle atrophy [15].

Conservative treatments such as physiotherapy, acupuncture, aquatic and specific exercise programs, and maternity support belts have all been described in the research literature as interventions of some benefit during pre- and postnatal phases [16]. Systematic reviews by Ho et al. (2008) [17] and Richards et al. (2012) [18] indicated that support belts assist with pain or reduction of symptoms; however, compliance in wearing maternity support belts is reported to be poor due to reduced comfort when wearing the garment, lack of ease of use, reduced aesthetics, concerns about skin comfort (i.e., heat and excessive pressure) [19], and impact on kinematic changes during pregnancy [20]. Depledge et al. (2005) [21] found that maternity support belts did not enhance the effects of advice and exercise on the management of symphysis pubis dysfunction, but a study using cadavers determined that there was an increase in stabilization of the sacroiliac joints from the mechanical effects of rigid maternity support belts providing consistent compression [22]. Furthermore, previous research in this field has suggested that prenatal and postnatal complications require active interventions and that a holistic and multimodal intervention approach has demonstrated the greatest effectiveness in relieving symptoms [3,23].

One component of a multimodal approach may be the use of dynamic elastomeric fabric orthoses (DEFO) or compression garments [19,24,25,26]. Commercial manufacturers have used previously reported research on this topic to design specific compression garments that target women in the prenatal and the postnatal periods with the objective of increasing wear-compliance based on the improved aesthetics and comfort of the DEFO garments in addition to their targeted design to provide support during functional activity. DEFO garments differ in design, material, and compression grades to support belts. Sawle et al. (2016) [26] discussed the concept of DEFO in pregnancy and postpartum as an intervention tool to manage common musculoskeletal conditions. Specifically, DEFO are thought to address common musculoskeletal conditions in women during pre and postnatal periods through a consistent applied force from customized and precisely positioned elastomeric panels that stabilize joints around the pelvic girdle, allowing for increased function whilst wearing the DEFO garment [26]. However, research examining the effectiveness, the feasibility, and the acceptability of DEFO to achieve these outcomes during pregnancy and in the postnatal phase is limited.

Close (2016) [5] suggested that women during pregnancy and in the postpartum period receive inadequate guidance from healthcare professionals in relation to managing common prenatal and postnatal ailments. Research suggests that this may be due to the limited empirical evidence available regarding the most appropriate interventions for women in prenatal and postnatal periods to reduce pain and maintain daily activity [5,27,28,29,30].

As maternity care providers are being encouraged to utilize more evidence-based practice to provide high quality interventions for their patients, it is imperative that further research be conducted on non-pharmacological intervention strategies to evaluate their effectiveness for reducing pain and increasing functional capacity in prenatal and postnatal care.

On this basis, the aims of this systematic review are to: i) determine the effectiveness of dynamic fabric orthoses (DEFO) for managing pain, improving functional capacity, and enhancing quality of life in prenatal and postnatal phases of care; and ii) assess the feasibility and the acceptability for women of using DEFO in prenatal and postnatal phases of care.

## 2. Materials and Methods

The procedures used in this systematic review were guided by the third edition of the Centre for Reviews and Dissemination (CRD) guidance for undertaking systematic reviews in healthcare [31] and informed by the Preferred Reporting Items for Systematic Reviews and Meta-Analysis (PRISMA) statement [32].

### 2.1. Identification of Studies

To identify relevant research studies for this systematic review, a literature search was performed on 2 April 2019. The databases searched included PubMED, Cumulative Index to Nursing and Allied Health (CINAHL), EMBASE, Cochrane, and Physiotherapy Evidence Database (PEDro). Additional manual searches were completed of the reference lists of all full-text articles that were included in the review as well as systematic reviews that were identified during the search. Key terms were identified for the search, including “compression garment”, “compression stocking”, pelvic support belt, prenatal, postnatal, and synonyms for each of these search terms. The search strategy was modified for each individual database and included appropriate truncations, application of “wild cards” when able, appropriate use of Boolean Operators to combine the key search terms, and setting of filters where available to collect the most current evidence from full text journals. The comprehensive and customized search strategy used for each electronic database is available in Table S1.

### 2.2. Screening and Selection Procedures

The literature search results were imported into EndNote X8 [33], and duplicate articles were removed. An initial screening process reviewing titles and abstracts was undertaken to identify possibly relevant papers, and any papers clearly not meeting eligibility for inclusion were removed. The next step involved review of the remaining articles in full text, applying the predetermined inclusion and exclusion criteria defined below. To be included in this review, studies were required to adhere to the following inclusion criteria:

1. Full text articles published in English or articles that could be translated into English.
2. Published between January 2000 and April 2019.
3. Constituted a report of a study with one of the following designs: randomized controlled trials, clinical controlled trials, prospective cohort studies, quasi-experimental studies, pilot studies, cross-sectional studies, single case studies.
4. Study participants included women between the ages of 18–50 of any race or socioeconomic status in specified prenatal or postnatal periods as follows. Specifically, in prenatal studies, women were to be between 12–40 gestational weeks, and in postnatal studies, the study population needed to be immediately and/or up to 12 months postpartum.
5. Investigated the effectiveness, the feasibility, or the acceptability of DEFO/compression garments for addressing physiological, psychological, and/or social outcomes including pain (pelvic, low back/lumbar or vulval regions), functional capacity, and quality of life.
6. Compression garment location, style, and grade must include continuous contact with the torso and/or the pelvic region and the genital region, the perineum, and the thigh to be consistent with a DEFO.

The following three exclusion criteria were also applied:

1. Studies investigating women in the prenatal phase who experienced: Rectus Diastasis ≥6 cm, complications or co-morbidities such as preeclampsia, eclampsia, venous thrombus/deep vein thrombosis, thrombophlebitis, bleeding of the varicose vein, pulmonary embolism, vaginal/rectal prolapse, or an intellectual or mental impairment.
2. Studies investigating women in the postnatal phase who experienced: wound infection, severe haemorrhaging, infection, Rectus Diastasis ≥6 cm, complications or co-morbidities such as preeclampsia, eclampsia, venous thrombus/deep vein thrombosis, thrombophlebitis, bleeding of the varicose vein, pulmonary embolism, vaginal/rectal prolapse, or an intellectual or mental impairment.
3. Studies investigating women who had surgical management other than caesarean section or episiotomy in prenatal or postnatal care.

Studies that were eligible for review were divided into two categories: “prenatal care” and “postnatal care” studies. Manual searches of the reference lists of included studies were conducted to identify any additional studies that could be included in this review that were not identified in the original search, and inclusion and exclusion criteria were applied to those studies as well to determine their eligibility for inclusion.

### 2.3. Critical Appraisal of Methodological Quality

A modified version of the methodological quality checklist published by Downs and Black (1998) [34] was used to critically appraise both randomized controlled trials and other types of studies included in this review and to determine the methodological quality of each study. The Downs and Black (1998) [34] critical appraisal tool is a 27-point scale consisting of five subscales: (i) reporting, (ii) external validity, (iii) internal validity bias, (iv) internal validity confounding, and (v) power [34]. The Downs and Black scale has high internal consistency (*r* = 0.89) and criterion validity (*r* = 0.90), good test-retest reliability (*r* = 0.88), and inter-rater reliability (*r* = 0.75) [35]. For the purpose of this systematic review, a modification was made in relation to the scoring of power following assessment of whether a power or sample size calculation was reported in each included study or not (Yes = 1, No/Unable to determine = 0, Partially = 0). This modification is consistent with modifications previously reported by Jäkel et al. (2011) [36]. The studies were rated as being of poor methodological quality if they scored 7 or less, limited quality if they scored 7–13, moderate quality if they scored 14 to 20, and strong quality if they scored 21 or greater [36]. The quality of the included studies was considered in the synthesis of the results; however, studies were not excluded if they were of poor quality.

The methodological quality assessment for the included full-text studies was independently conducted by two reviewers (J.S., N.M.). Post independent appraisal, the results were discussed and consensus was reached between the two reviewers to resolve any discrepancies. Cohen’s kappa (κ) was run to assess the level of concordance between the two reviewers’ appraisals. The results of the Cohen’s κ analysis can be interpreted as follows: values ≤ 0 indicate no agreement, 0.01–0.20 as no to slight agreement, 0.21–0.40 as fair agreement, 0.41–0.60 as moderate agreement, 0.61–0.80 as substantial agreement, and 0.81–1.00 as almost perfect agreement [37].

### 2.4. Data Extraction

Data extraction was based on the Cochrane Consumers and Communication Review Group data extraction template [31]. The categories of data extracted from the studies were study design, country where study took place, environment/setting of the study, study aims, participant characteristics (number included in study population, age, gestational week, number of pregnancies, etc.), pathologies (deep vein thrombosis (DVT), musculoskeletal, common ailments, lymphedema, etc.), interventions used (physiotherapy, alternative therapies, support/compression garments, medicinal, exercise, forceps, etc.), primary and secondary outcome measures (pain scales, functional capacity questionnaires, health surveys, etc.), main findings, and conclusions. Extracted data were tabulated to enable ready comparison of findings from the included studies.

### 2.5. Synthesis

Once data were extracted from the included studies, a critical narrative synthesis approach was utilised to: 1) summarise and critique the methodologies of the included studies, and 2) to compare and contrast the findings from included studies with focus on findings relevant to the aims of the present review.

Additionally, a meta-synthesis was conducted with included studies using methods previously published to evaluate the main themes concerning the effectiveness of using DEFOs for managing pain, improving functional capacity, and enhancing quality of life [38]. The meta-synthesis was conducted across three rounds to ensure that all published emperical literature was investigated while allowing conclusions to be drawn from higher methodological quality studies: (i) all included prenatal and postnatal studies; (ii) included prenatal and postnatal studies with moderate to strong methodological quality [34,36]; and (iii) included randomised control trials with moderate to strong methodological quality [34,36]. To determine the summary of the DEFO effectiveness for each variable, the following criteria were applied: (i) “no effect” was coded (0) if 0–33% of investigations reported no significant effect; (ii) uncertain was coded (?) if 34–59% reported a significant effect or less than five investigations reported on the variable; and (iii) depending on the direction of the effect, a desirable effect was coded (+) or an undesirable effect was coded (−) if ≥60% of investigations reported a significant effect [38,39].

## 3. Results

### 3.1. Included Studies and Participant Characteristics

**Figure 1:**
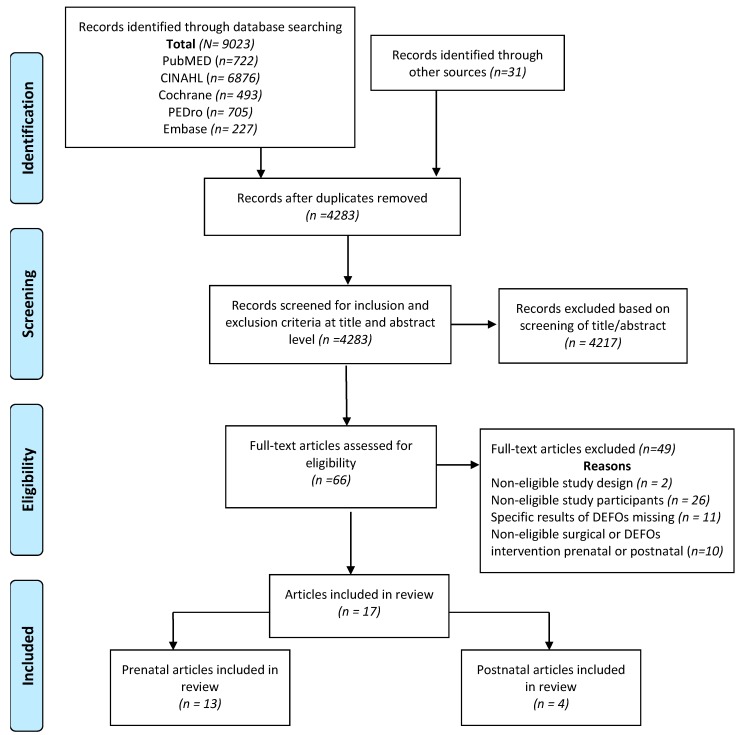
Preferred Reporting Items for Systematic Reviews and Meta-Analyses (PRISMA) flow diagram [32] depicting results of the systematic search, screening, and selection processes.

The initial literature search yielded 9023 studies, and the subsequent manual search of the reference lists from included studies identified 31 further potentially eligible studies. The results of subsequent stages of the screening and selection processes are depicted in the PRISMA flow diagram [32] in Figure 1 and ended in identification of 17 eligible studies for inclusion in the review (Table S2). Of these studies, 13 reported outcomes for women in the prenatal stages, and four reported outcomes for women in the postnatal stages.

The 17 studies that were reviewed based on the eligibility criteria varied in design (Table S2). Ten studies were randomized controlled trials [21,25,40,41,42,43,44,45,46,47], one was a randomized crossover trial [48], two studies were randomized pilot studies [49,50], two were quasi-experimental studies [20,51], and there was one of each of the following designs—a prospective cohort study [24] and a case control study [52]. The studies were also diverse in geographical locations, which included (Table S2): New Zealand (*n* = 2) [21,50], United States of America (*n* = 2) [47,49], Iran (*n* = 2) [44,46], Germany (*n* = 3) [40,42,48], The Netherlands (*n* = 2) [20,52], Australia (*n* = 1) [25], Belgium (*n* = 1) [41], England (*n* = 1) [43], Scotland (*n* = 1) [51], Turkey (*n* = 1) [24], and Sweden (*n* = 1) [45].

The included studies together involved 1251 participants. The mean age of the female participants was 30.0 years, excluding the studies by Kalus et al. (2007) [25], Jamieson et al. (2007) [51], and Carr (2003) [49], who did not report participant ages. Whilst Carr (2003) [49] did not report mean age of participants, the women in that study were noted to be 19 years or older. In twelve of the studies (Table S2), women were in the prenatal stages, varying in gestational weeks ranging from first trimester to delivery. Two studies focused on women during pregnancy until gestational week 36–38 and performed follow up evaluations in the postpartum period [43,45]. Three of the studies occurred strictly in the immediate postnatal phase following delivery [46,47,51], while one study [20] investigated women within 5 years postpartum. The included studies exhibited considerable heterogeneity in participant characteristics, gestational weeks, weeks postpartum, sample sizes, duration and frequency of interventions, and functional outcome measures, and thus a meta-analysis was not conducted. Rather, key data from included studies that informed this systematic review are presented in tabulated form in Table S2 and are narratively synthesized in the sections below.

### 3.2. DEFO Investigated in the Included Studies

**Figure 2:**
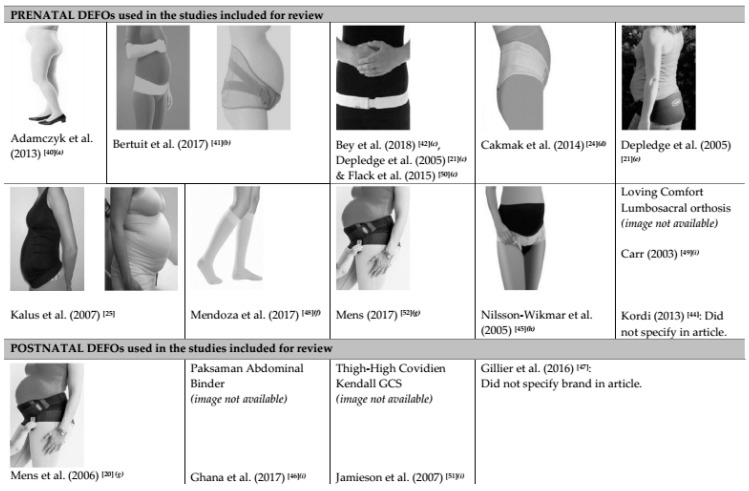
Different dynamic elastomeric fabric orthoses (DEFO) used in the studies included in this review. Images were reproduced with permission from: ^(a)^ “Venotrain^®^ micro ATU”, https://www.kompressionsstruempfe.ch/venotrain-micro/119-atu-schwangerschaft.html. © 2017 Kompressionsstruempfe.ch; ^(b)^ “Thuasne A) Ortel-P”, https://www.thuasne.com/en/all-products/back/ortelr-p B) “Thuasne LombaMum”, https://www.thuasne.com/en/all-products/back/lombamumr. Copyright www.thuasne.com; ^(c)^ “LifeCare Symphysis Pubis Belt”, https://www.orthotics.co.nz/our-products/, © Copyright Orthotics Centre 2019. ^(d)^ “Variteks Ortopedi—Elastic Pregnancy Corset”, http://www.variteks.com/en/product/code-139-elastic-pregnancy-corset/. Copyright © Variteks 2017; ^(e)^ “Smiley Belt”, http://www.smileybelt.co.nz/blog/why-wear-a-flexible-pregnancy-support-belt. © Copyright SmileyBelt 2019; ^(f)^ “SIGVARIS Cotton CCL1, CCL 2, CCL 3”, https://www.sigvaris.com/global/en/product/cotton-0. © 2017 SIGVARIS; ^(g)^ “Rafys Belt”, Mens (2017) [50] and Mens et al. (2006) [18], https://www.rafys.nl/index.php/productdetails/1053/rafys-bekkenbrace-sisda-meegroeiband-15-cm; ^(h)^ “Rehband Sacroiliac belt”, https://cosmac.com.au/Rehband-1045-dosi-sacro-belt.html © 2014 Cosmac Healthcare; ^(i)^ Image not available—due to copyright.

All intervention studies examined some type of maternity support or DEFO (Table S2). Across the included studies, the garments varied greatly in design, compression classification, materials of which they were constructed, and location that the garment was worn. Two studies [44,47] did not clearly describe or identify the DEFO used, therefore limiting the reproducibility of their methods in subsequent research. Figure 2 provides a visual representation of the different of the DEFOs worn during the included studies. The interventions are detailed in Table S2 showing varying names, styles, and positions of the maternity support/DEFO interventions.

### 3.3. Ailments that DEFOs were Used to Treat during Prenatal and Postnatal Care

Eight of the prenatal studies included in this review focused on complaints of low back or pelvic pain as the primary ailment, while others examined chronic venous insufficiencies, nauseas and vomiting, and dynamic and postural stability. The postnatal studies contained in this review concentrated on ailments such as wound healing, pain levels following caesarean sections, pregnancy-related pelvic gridle pain continued during the postnatal phase, and postnatal venous thromboembolism of the common femoral vein (Table S2).

### 3.4. Methodological Quality of Included Studies

Three of the seventeen studies were deemed to be of limited methodological quality, twelve of the studies were deemed to be of moderate methodological quality, and two studies were assessed to be of strong methodological quality (Table S2). Total scores for the included studies in this review ranged from 11 to 23 out of a possible 27 points. The Cohen’s Kappa (κ) analysis indicated substantial agreement between the authors (JS and NM) who assessed the methodological quality of included studies (κ = 0.736, *p* < 0.0001). Consensus was reached between the two reviewers in each instance where a score difference was observed, and a further reviewer was not required to resolve differences.

The main limitations of the included studies were reporting bias such as not identifying confounding factors, not commenting on adverse events, reduced external validity, internal validity bias, and how power was determined to detect a clinically important effect and how the sample sizes had been calculated.

Overall methodological strengths of included studies were the reporting of study aims and/or objectives, identifying the main outcomes to be measured as being valid and reliable, detailing the interventions of interest, and the main findings of the study being clearly described.

### 3.5. Aim 1 (Prenatal): Effectiveness of Using DEFO in Prenatal Care to Manage and/or Improve Pain, Functional Capacity, and Quality of Life

#### 3.5.1. Effectiveness of Using DEFO in Prenatal Care to Manage and/or Improve Pain

Ten [21,25,41,43,44,45,49,50,52] out of the thirteen included prenatal studies reported on the effectiveness of maternity support and/or lumbopelvic belts in the prenatal period to manage or improve pain. Of the seven studies that reported significant differences in pain, four reported that pain was significantly decreased within the groups wearing the DEFO where participants reported fewer days or hours of pain [41,44,49,50], and one study reported significantly less impact of pain on functional variables [25]. One study found that those participants that wore a pelvic belt during hip adduction movements were able to achieve a higher adduction force with less reported pelvic girdle pain than those without a belt (*p* < 0.001) [52]. A more recent study showed in its prenatal data analysis that pain was reduced more in the group wearing a customized DEFO when compared to an off-the-shelf rigid pelvic belt, demonstrating significant differences between groups (*p* < 0.05) during both the day and the night [43] (Table S2). A meta-synthesis of findings regarding the effectiveness of using DEFOs suggested a desirable effect for managing pain during prenatal care (Table 1).

Conversely, four studies indicated no significant differences in pain for women who wore the support belts/garments compared to those who did not. Of these four studies, two studies found significant average pain reductions in both the control group (i.e., exercise group only or tubigrip) and the intervention groups (i.e., rigid or non-rigid belt), but no add-on effects were demonstrated with the addition of a nonrigid or a rigid belt [21,25], and three studies reported no observed significant differences amongst the treatment groups [25,41,45] (Table S2).

#### 3.5.2. Effectiveness of Using DEFO in Prenatal Care to Manage and/or Improve Functional Capacity

Eight [21,24,41,42,43,44,45,50] out of the thirteen included prenatal studies examined the changes on functional capacity when wearing the maternity support belts or DEFO. Three studies reported that the use of pelvic belts to perform daily tasks caused a decrease in pain and improved ability to perform daily activities such as standing, sitting, rolling over in bed, and walking [21,41,44]. Two studies demonstrated that maternity support belts significantly improved women’s postural stability [24,42], and as a result, those individuals with a maternity support belt had lower falls risk [24]. Two studies reported that, when comparing time of inclusion scores to end of study scores in the postpartum period, all groups (i.e., wearing rigid or non-rigid pelvic belt or DEFO) scored significantly higher (i.e., better) activity ability [42,45].

Two studies reported that there was no significant difference at follow-up between the two groups of women using a maternity support belt (i.e., rigid belt or non-rigid belt or customized DEFO), reporting similar activity levels between the groups [43,50]. However, the two studies observed that the scores improved when wearing pelvic belts or DEFO [43,50] (Table S2). Table 1 provides findings of a meta-synthesis demonstrating a significant desirable effect for improving functional capacity when women wore DEFOs during pregnancy.

#### 3.5.3. Effectiveness of Using DEFO in Prenatal Care to Manage and/or Improve Quality of Life

Six [25,40,43,44,48,49] out of the thirteen prenatal studies used a health survey to explore the impact of DEFO on the participants’ psychological well-being and to determine the overall satisfaction/concerns for its use. Four studies reported that participants who were in the intervention group reported a significant improvement on the quality of life questionnaire, specifically reporting greater satisfaction with treatment, better scores in the mental health components of the questionnaire, and less burden imposed by the treatment [40,43,44,48], with one study showing significantly better reported results in all components of quality of life except for social relation when the DEFO group was compared to the other groups [44].

Contrary to the findings outlined above, three studies found no significant differences from preintervention to postintervention for either group [25,43,49] or significant differences between the support device groups [25,43] (Table S2).

The meta-synthesis of studies with moderate to strong methodological quality suggested that wearing DEFOs improved quality of life for women in the prenatal period; however, this desirable effect was not evident when the meta-synthesis included randomized controlled trials (RCT) only (Table 1).

### 3.6. Aim 1 (Postnatal): Effectiveness of Using DEFO in Postnatal Care to Manage and/or Improve Pain, Functional Capacity, and Quality of Life

#### 3.6.1. Effectiveness of Using DEFO in Postnatal Care to Manage and/or Improve Pain

One study out of the four included postnatal studies reported that the use of DEFO resulted in significant decreases in the amount of administered analgesic medications in the immediate postnatal period for pain for females that wore the DEFO compared to those that did not [46]. Conversely, another study demonstrated no significant decreases in pain postpartum when female participants wore DEFO and/or pelvic belts [47] (Table 1 and Table S2).

#### 3.6.2. Effectiveness of Using DEFO in Postnatal Care to Manage and/or Improve Functional Capacity

Three [20,46,51] out of four included postnatal studies observed positive significant changes in the impact of DEFO and/or compression stockings on function postpartum in areas such as wound edge healing [46], a decrease in the common femoral vein diameter [51], and significantly less sacroiliac joint laxity [20] (Table 1 and Table S2).

#### 3.6.3. Effectiveness of Using DEFO in Postnatal Care to Manage and/or Improve Quality of Life

One study by Ghana et al. (2017) [46] used a health survey and Patient Satisfaction questionnaire five days following caesarean section to explore the impact of DEFO on the participants’ psychological well-being and to determine the overall satisfaction/concerns for its use. Ghana et al. (2017) [46] did not find a significant difference between the intervention group (wearing an abdominal binder continuously for 14 h) and the control group (no abdominal binder) in Patient Satisfaction when measured on a 5-point Likert Scale (*p* = 0.443) (Table 1 and Table S2).

The meta-synthesis of studies with moderate to strong methodological quality found an inadequate number of investigations to demonstrate an overall effect from wearing DEFOs during the postnatal period for decreasing pain, improving functional capacity, or enhancing quality of life (Table 1).

### 3.7. Aim 2: Feasibility, and Acceptability of Using DEFO in Prenatal and Postnatal Care

#### 3.7.1. Feasibility and Acceptability of Using DEFO in Prenatal Care

None of the included prenatal studies reported adverse events or that there were no adverse events to report. Overall, loss of participants in the prenatal studies were largely due to pre-term delivery, pregnancy-related complications (i.e., placenta abruption), non-compliance with follow-up appointments or completion of questionnaires, and/or personal reasons [21,24,25,40,44,45,48,49,50,52].

Five [21,41,48,49,50] out of thirteen prenatal studies included in this review discussed aspects of adherence to use of maternity support belts and/or DEFO. All five studies found women in the studies reported a high level of acceptability and satisfaction with the pelvic belt [21,41,48,49,50], found the use of the pelvic belt to be excellent [21,41,48,49,50], reported the belt to be comfortable to wear [49,50], and reported high levels of compliance for wearing the DEFO [21,48].

Two studies observed a small percentage of women in the intervention group who reported that they were not satisfied [49,50]. However, the participants were reported to have addressed any minor irritations (i.e., belt “rides up” and with sitting, “digging in”) independently (i.e., wearing longer t-shirts and/or high-rise underwear or briefs) and concluded that the positive impact of the belt on the main complaints outweighed these minor annoyances [49,50].

All five studies showed that the intervention group reported compliance and did not report any concerns that would have caused cessation of the DEFO, as there were no alterations in skin integrity reported by participants or confirmed by a physical examination [21,41,48,49,50].

#### 3.7.2. Feasibility and Acceptability of Using DEFO in Postnatal Care

Two [46,47] out of four included postnatal studies reported on adverse events. Gillier et al. (2016) [47] reported that no adverse events in their study were observed with the use of abdominal binders, whereas Ghana et al. (2017) [46] reported the loss of two participants in the intervention group because of abdominal binder intolerance and hemorrhaging. Overall, the loss of participants in the included postnatal studies was primarily due to non-compliance with follow-up appointments or completion of questionnaires and/or private and undisclosed personal reasons [20,46,47,51].

Three [46,47,51] postnatal studies discussed the concept of acceptability of DEFO postpartum. Ghana et al. (2017) [46] indicated that only two out of 178 participants reported abdominal binder intolerances in the intervention group and stopped its use but did not report further on acceptability among the remaining 176 participants in the intervention group. Gillier et al. (2016) [47] reported the positive impact of wearing an abdominal binder on pain and distress levels for participants in the immediate post-caesarean section period with the potential to decrease the development of chronic pain postoperative. Jamieson et al. (2007) [51] suggested the wearing of graduated compression stockings was recommended in the reduction of thrombosis; however, a large scale randomized clinical trial would be recommended to provide strong evidence for its use and feasibility.

## 4. Discussion

### 4.1. Overview of Findings

The primary aim of this systematic review was to critically assess whether DEFOs are effective in managing and/or improving pain, functional capacity, and/or quality of life in prenatal and postnatal phases of care. Additionally, the review aimed to assess if DEFOs were feasible and acceptable to wear in prenatal and postnatal phases of care. To address these aims, this systematic review identified seventeen studies of varying designs that investigated the use of DEFOs as an intervention for managing and/or improving common musculoskeletal ailments during prenatal and postnatal care. This review identified concerns surrounding the heterogeneity and the methodological quality of the included studies, such as study design, variability in DEFO, differing participant characteristics (e.g., gestational weeks, parity), functional outcome measures used, and applied interventions, which are consistent with the conclusions by Ho et al. (2009a) [2], Gutke et al. (2015) [53] and Richards et al. (2012) [18]. The above limitations prevented the execution of a meaningful meta-analysis. Therefore, a meta-synthesis of the findings from all prenatal and postnatal studies was undertaken to assist in concluding on the effectiveness of DEFOs. In relation to our primary aims, the meta-synthesis revealed an overall desirable effect of wearing DEFOs for managing pain and improving functional capacity during pregnancy. There is not currently sufficient evidence to recommend the use of a DEFO for women during pregnancy to enhance their quality of life. Further, there is a lack of evidence to support the use of a DEFO in the postnatal period for managing pain, improving functional capacity, or enhancing quality of life at this time.

### 4.2. Aim 1: Effectiveness of Using DEFO’s in Prenatal and Postnatal Care to Manage and/or Improve Pain, Functional Capacity, and Quality of Life

The review found that DEFOs may have a role in the reduction of back pain in activities of daily living such as sleeping, walking, standing, and getting up from a sitting position [25,40,41,44,45,50]. Whilst limited, the evidence found in this review is consistent with that from previous reviews [2,18,53] suggesting that DEFO can be effective in the reduction of pain in relation to common ailments during prenatal phases of care, but insufficient evidence was available to support this finding in postnatal phases of care. This review demonstrates similar findings to the systematic review by Gutke et al. (2015) [53], where the authors reported that pelvic belts significantly reduced lumbopelvic pain.

Improving functional capacity is an important outcome when considering the use of DEFO in prenatal and postnatal populations, as this is one of the primary complaints reported by women—reports of interference with activities of daily living such as sleep, work, standard daily activities, and balance are common [2,24,54,55]. Typical physiological changes during pregnancy result in altered biomechanics and balance, placing pregnant women at an increased risk of falls with a significant increase in risk occurring in the third trimester [24]. There was some evidence in this review to suggest that an improvement in stabilization and balance could be observed in pregnant women when using DEFOs, enabling women to be both safer and more capable in everyday function. However, it is important to note that this conclusion should be taken more as an observation or suggestion, as the methodological weakness across the literature in this field is apparent, with various DEFOs being utilized for the intervention, limited description of prescribed exercises, lack of blinding the participants and/or the therapist, inadequate sample sizes, and the use of outcome measures where reliability and validity have not been substantiated. These conclusions are consistent with more recent reviews by Richards et al. (2012) [18] and Gutke et al. (2015) [53], noting that further research has been completed in the area of the biomechanics and the physiology of pelvic belts on the lumbopelvic region, as suggested in the review by Ho et al. (2009a) [2].

Based on the results of quality of life questionnaires/health surveys completed by participants in this review, wearing DEFO resulted in a high level of treatment satisfaction and improved quality of life [40,41,44,48]. Interestingly, although studies reported a decrease in pain or improvement in functional capacity when wearing DEFO, not all studies that reviewed quality of life found a significant difference [25,46,49]. Literature that reported significant improvement on quality of life questionnaires concluded that there was an improvement in the quality of life for those participants that received DEFO even if improvement was not observed for pain or functional capacity [40,44,48]. The limitation in comparing the literature to draw conclusions or suggest clinical implications is the inconsistency in the use of quality of life questionnaires, which varied throughout the literature. Previous reviews by Ho et al. (2009a) [2], Gutke et al. (2015) [53], and Richards et al. (2012) [18] did not investigate the impact DEFO has on the participants quality of life questionnaires/health surveys; therefore, similarities or comparison to the present review cannot be made.

Overall, the meta-synthesis suggested that DEFOs have a significant desirable effect in prenatal care for managing pain and improving functional capacity. Whilst the effects of DEFOs for enhancing quality of life in prenatal care appear promising, further research using robust methodologies such as RCTs is needed. There is some evidence to suggest that DEFOs may improve functional capacity in postnatal care. However, studies using more robust methodologies are scarce, supporting the need for further RCTs in this area (Table 1).

### 4.3. Aim 2: Feasibility and Acceptability of Using DEFOs in Prenatal and Postnatal Care

This review found evidence to suggest that pelvic belts can lead to skin comfort concerns and discomfort while wearing the DEFO—particularly during pregnancy. However, other included studies did report them to not be present or to be only minimally present with participants who reported the minor annoyances and who were able to find strategies to minimize these annoyances. These findings are similar to those found in the review by Ho et al. (2009a) [2], suggesting that some adverse effects may be present when women wear the DEFO during pregnancy. Suggestions have been made in the included studies of the present review that the use of abdominal binders in the postnatal period can decrease hospital length of stay. However, as there are presently no review studies for women in the postpartum period examining the effects, the feasibility, and the acceptability of using DEFOs or the emotional and the psychological effects, this review cautions that generalizability is minimal due to the limited evidence and suggests that large randomized control trials are needed to be more conclusive. Therefore, further research is required to make sound clinical decisions regarding the prescription of DEFOs, as the effectiveness, the feasibility, and the acceptability of compression garments during pregnancy are not yet confirmed in the literature.

### 4.4. Strengths and Limitations of the Review

The present review has strength in its thorough search strategies, its systematic nature, the use of a validated critical appraisal tool, and a meta-synthesis of results. The heterogeneity in designs, outcome measures, and DEFO garments investigated across the included studies reduces the strength of findings and the external validity of this review. However, the main limitation to this review is the limited evidence and research available for the populations of interest. As indicated, there are few published studies exploring the effectiveness of DEFO during postnatal care.

## 5. Conclusions

Although seventeen studies were included in this review, which examined a DEFO as an intervention during prenatal and postnatal phases, to date, there is still little high-quality evidence to support the use of DEFO in prenatal and postnatal populations. Small study samples, inconsistent use of reliable and valid outcome measures, and varied definitions of a DEFO and/or maternity support belts have all contributed to the lack of high-quality empirical studies on this topic. The meta-synthesis conducted in the present review suggests that, during pregnancy, wearing a DEFO can have a desirable positive effect for managing pain and improving functional capacity. However, there is limited evidence available to suggest that wearing a DEFO during pregnancy can affect quality of life. More research is required to determine the clinical relevance of wearing a DEFO for women in the postnatal period. Future research in this field should include standardized outcome measures, standardized criteria for DEFO, accurate product descriptions, and high-quality study designs so that valid conclusions can be drawn and, where applicable, research evidence can be implemented in clinical practice.

### Clinical and Research Implications

This systematic review indicates that there is high-quality evidence to support the value of DEFOs in prenatal period for managing pain and improving functional capacity. This gap in the literature regarding the use of DEFOs in the postnatal population does not lead us to conclude that their use is not warranted or effective but rather suggests further research is required to examine the effectiveness of DEFO in postnatal populations and assist clinicians in establishing evidence-based practice in this women’s health field.

## Figures and Tables

**Table 1:** Meta-synthesis of reported findings for included prenatal and postnatal studies on the effectiveness of using DEFO for the management of pain, improving functional capacity and enhancing quality of life.

All Studies Included				
Variable	Significant Effect *n*	Non-Significant Effect *n*	Summary of Coding	
			*n*/*N*^a^ (%) for Variable	DEFO ^b^ Effect (+/−,0,?) ^c^
Prenatal Studies				
Pain	9 [21,25,41,43,44,45,49,50,52]	0	9/9 (100.0)	+
Functional Capacity	15 [21,24,25,40,41,42,43,44,45,49,50,52]	3 [24,42,43]	15/18 (83.3)	+
Quality of Life	7 [40,43,44,48]	1 [25]	7/8 (87.5)	+
Postnatal Studies				
Pain	2 [46,47]	0	2/2 (100.0)	?
Functional Capacity	5 [20,46,51]	1 [46]	5/6 (83.3)	+
Quality of Life	2 [46,47]	0	2/2 (100.0)	?
Studies of Moderate to Strong Quality Included Only ^d^				
Variable	Significant Effect *n*	Non-Significant Effect *n*	Summary of Coding	
			*n*/*N*^a^ (%) for Variable	DEFO ^b^ Effect (+/−,0,?) ^c^
Prenatal Studies				
Pain	9 [21,25,41,43,44,45,49,50,52]	0	9/9 (100.0)	+
Functional Capacity	11 [21,25,41,42,43,44,45,49,50,52]	2 [42,43]	11/13 (84.6)	+
Quality of Life	6 [43,44,48]	1 [25]	6/7 (85.7)	+
Postnatal Studies				
Pain	2 [46,47]	0	2/2 (100.0)	?
Functional Capacity	2 [46,51]	1 [46]	2/3 (66.7)	?
Quality of Life	2 [46,47]	0	2/2 (100.0)	?
Randomised Control Trials Included Only				
	Significant Effect *n*	Non-Significant Effect *n*	Summary of Coding	
			*n*/*N*^a^ (%) for Variable	DEFO ^b^ Effect (+/−,0,?) ^c^
Prenatal Studies				
Pain	6 [21,25,41,43,44,45]	0	4/6 (66.7)	+
Functional Capacity	7 [21,25,41,42,43,44,45]	2 [21,43]	7/9 (77.7)	+
Quality of Life	3 [40,43,44]	1 [25]	3/4 (75.0)	?
Postnatal Studies				
Pain	2 [46,47]	0	2/2 (100.0)	?
Functional Capacity	1 [46]	1 [46]	1/2 (50.0)	?
Quality of Life	2 [46,47]	0	2/2 (100.0)	?

^a^*n*/*N* for Outcome (%): *n* = significant positive effect divided by *N* = all studies that provide statistical evidence. ^b^ DEFO—dynamic elastomeric fabric orthoses. ^c^ Effect (+/−, 0, ?): no effect (0) = 0–33%; uncertain (?) = 34–59% or less than five investigations reported on variable; desirable effect (+) or undesirable effect (−) = ≥60%. ^d^ Critical appraisal using a modified version of the Downs and Black (1998) methodological quality checklist [34]; study scored of moderate quality (scored between 14 to 20) or strong quality (scored 21 or greater) [36].

## Supplementary Materials

The following are available online at https://www.mdpi.com/1660-4601/16/13/2408/s1. Table S1: Comprehensive and customized search strategy used for each electronic database. Table S2: Study design & aims, participant characteristics and key data extracted, outlining the interventions, functional outcome measures, main findings and conclusions of included studies.
