# Supplementary material for: Effectiveness, Feasibility, and Acceptability of Dynamic Elastomeric Fabric Orthoses (DEFO) for Managing Pain, Functional Capacity, and Quality of Life during Prenatal and Postnatal Care: A Systematic Review
Source: Int J Environ Res Public Health. 2019 Jul 6;16(13):2408. doi: 10.3390/ijerph16132408 (PMC6651323; doi:10.3390/ijerph16132408)
Supplement: Supplementary file 1 [file ijerph-16-02408-s001.zip › Table S1_Comprehensive and customized search strategy used for each electronic database.docx]

**Table S1: Search Results from Different Electronic Databases**

| **Database** | **Search terms** | **Results** |
| --- | --- | --- |
| PubMED | (((Compression AND (garment* or therap* or stock* or hosier* or short* or corset* or orthos*)) or dynamic elastomeric fabric orthos* or pelvic belt* or maternity belt* or support belt* or support garment* or "Stockings, Compression"[Mesh]) AND ((Pregnan* or "Pregnancy"[Mesh] or prenatal or maternity or "Maternal Health Services"[Mesh] or antenatal or "Perinatal Care"[Mesh] or perinatal or postnatal or postpartum) OR ("Delivery, Obstetric"[Mesh] or Caesarean or cesarean or c-s or c-section or perineal or episiotomy or vaginal deliver* or vaginal birth* or normal deliver* or normal birth* or NVD))) | 722 |
| CINHAL | (((Compression AND (garment* OR therap* OR stock* OR hosier* or short*or or corset or orthos*)) or dynamic elastomeric fabric orthos*)) OR "pelvic belt*" OR "maternity belt*" OR "support belt*" OR "support garment*" OR (MH "Compression Garments") OR (MH "Compression Therapy")) AND ((Pregnan* OR (MH "Pregnancy+") OR prenatal OR maternity OR antenatal OR perinatal OR postnatal OR postpartum) OR ((MH "Delivery, Obstetric+") OR Caesarean OR cesarean OR c-s OR c-section OR perineal OR episiotomy OR "vaginal deliver*" OR "vaginal birth*" OR "normal deliver*" OR "normal birth*" OR NVD))) | 6,876 |
| Cochrane | (((Compression AND (garment* OR therap* OR stock* OR hosier* or short* or corset or orthos*)) or dynamic elastomeric fabric orthos* OR "pelvic belt*" OR “maternity belt*" OR "support belt*" OR "support garment*" OR [mh “Stockings, Compression”])) AND (((Pregnan* OR [mh Pregnancy] OR prenatal OR maternity OR antenatal OR [mh “Perinatal Care”] OR perinatal OR postnatal OR postpartum)) OR (([mh "Delivery, Obstetric] OR Caesarean OR cesarean OR c-s OR c-section OR perineal OR episiotomy OR "vaginal deliver*" OR "vaginal birth*" OR "normal deliver*" OR "normal birth*" OR NVD))) | 493 |
| EMBASE | (((Compression NEXT/1 (garment* OR therap* OR stock* OR hosier* OR short* OR corset OR orthos*)) OR dynamic elastomeric fabric orthos*)) OR “pelvic belt*” OR “maternity belt*” OR “support belt*” OR “support garment*” OR ‘Stockings, Compression’/exp AND ((Pregnan* OR ‘Pregnancy’/exp OR prenatal OR maternity OR antenatal OR perinatal OR postnatal OR postpartum) OR (‘Delivery, Obstetric’/exp OR Caesarean OR cesarean OR c-s OR c-section OR perineal OR episiotomy OR "vaginal deliver*" OR "vaginal birth*" OR "normal deliver*" OR "normal birth*" OR NVD)) | 227 |
| PEDro | Compression | 705 |
| **Total Number of citations retrieved** | | **9,023** |
